# Plasmacytoid Dendritic Cells as Cell-Based Therapeutics: A Novel Immunotherapy to Treat Human Immunodeficiency Virus Infection?

**DOI:** 10.3389/fcimb.2020.00249

**Published:** 2020-05-26

**Authors:** Renée M. van der Sluis, Johanne H. Egedal, Martin R. Jakobsen

**Affiliations:** ^1^Aarhus Institute of Advanced Studies, Aarhus University, Aarhus, Denmark; ^2^Department of Biomedicine, Aarhus University, Aarhus, Denmark

**Keywords:** HIV, HIV latency, dendritic cells, DC vaccine, plasmacytoid DC, pDC, CD8+ T cells, NK cells

## Abstract

Dendritic cells (DCs) play a critical role in mediating innate and adaptive immune responses. Since their discovery in the late 1970's, DCs have been recognized as the most potent antigen-presenting cells (APCs). DCs have a superior capacity for acquiring, processing, and presenting antigens to T cells and they express costimulatory or coinhibitory molecules that determine immune activation or anergy. For these reasons, cell-based therapeutic approaches using DCs have been explored in cancer and infectious diseases but with limited success. In humans, DCs are divided into heterogeneous subsets with distinct characteristics. Two major subsets are CD11c^+^ myeloid (m)DCs and CD11c^−^ plasmacytoid (p)DCs. pDCs are different from mDCs and play an essential role in the innate immune system via the production of type I interferons (IFN). However, pDCs are also able to take-up antigens and effectively cross present them. Given the rarity of pDCs in blood and technical difficulties in obtaining them from human blood samples, the understanding of human pDC biology and their potential in immunotherapeutic approaches (e.g. cell-based vaccines) is limited. However, due to the recent advancements in cell culturing systems that allow for the generation of functional pDCs from CD34^+^ hematopoietic stem and progenitor cells (HSPC), studying pDCs has become easier. In this mini-review, we hypothesize about the use of pDCs as a cell-based therapy to treat HIV by enhancing anti-HIV-immune responses of the adaptive immune system and enhancing the anti-viral responses of the innate immune system. Additionally, we discuss obstacles to overcome before this approach becomes clinically applicable.

## Introduction

Antiretroviral therapy (ART) successfully suppresses human immunodeficiency virus (HIV) replication but it does not cure an individual from having the infection (Finzi et al., 1997). The major barrier to clearing the virus is the persistence of a latent reservoir in long-lived resting and proliferating memory CD4^+^ T cells (Chun et al., 1997; Wong et al., 1997; Hosmane et al., 2017). Different molecular mechanisms have been described that contribute to HIV latency, reviewed in Dahabieh et al. (2015) and Vanhamel et al. (2019). Accordingly, different strategies that aim at HIV clearance are investigated, reviewed in Margolis et al. (2020). One strategy is to induce HIV expression, combined with enhanced immune function to clear infected cells. Here, we hypothesize if therapeutic vaccination with pDCs can enhance immune function to clear HIV infected cells.

### Plasmacytoid DCs: A Small but Significant Cell Population

Dendritic cells (DCs) play a pivotal role in mediating innate and adaptive immune responses by various cellular mechanisms. In humans, DCs are categorized into heterogeneous subsets with distinct characteristics. Two major subsets are CD11c^+^ myeloid (m)DCs and CD11c^−^ plasmacytoid (p)DCs. Although both subsets are classified as DCs, they are in fact very different cell types. Initially, the primary role of pDCs was defined as type I Interferon (IFN)-producing cells in response to a viral infection (Cella et al., 1999; Siegal et al., 1999). PDCs were described as a cell type that effectively senses viral RNA or DNA via toll-like receptors (TLRs) 7 and 9 and subsequently produce vast amounts of type I and type III IFNs (Jarrossay et al., 2001; Kadowaki et al., 2001; Yin et al., 2012). However, the function of pDCs is more diverse, and amongst other functions, pDCs have been described to serve as antigen presenting cells (APC) that stimulate CD8^+^ and CD4^+^ T cells, or become “killer-pDCs” with cytotoxic properties, reviewed in Swiecki and Colonna (2015), Musumeci et al. (2019), and Reizis (2019).

DC-based vaccination has been of interest in the fields of oncology and infectious diseases for two decades. It is safe with few adverse effects but despite inducing favorable immune responses in preclinical studies, DC-based immunotherapies have not successfully induced significant clinical responses, reviewed in Mastelic-Gavillet et al. (2019) and da Silva et al. (2018). Because pDCs are key mediators for both innate and adaptive immune cells, interest in using pDCs for cell-based therapeutics is increasing. In this mini-review, we will discuss if therapeutic vaccination with pDCs could be beneficial for the treatment of HIV infection and focus on literature describing observations with pDCs. As the available studies are based on cells from several different species, we will try to simplify it by primarily referring to observations with human pDCs where possible. In doing so, we aim to answer the question: Can therapeutic vaccination with pDCs achieve a functional cure for HIV?

### pDC Vaccination in Humans

To this date, only two studies have tested peripheral blood-derived pDCs as cell-based cancer therapy. In the first trial, Tel et al., explored pDCs activated with inactivated Frühsommer-Meningoenzephalitis (FSME) and loaded with tumor antigen-associated peptides *ex vivo*, followed by administration to patients with metastatic melanoma (Tel et al., 2013a). The pDCs distributed over multiple lymph nodes (LNs), mounted both CD4^+^ and CD8^+^ T cell responses, and an IFN response was observed after each vaccination. This study showed that vaccination with pDCs was safe and induced favorable immune responses. The second study by Westdorp et al., investigated patients with chemo-naïve castration-resistant prostate cancer receiving vaccinations with mature CD1c^+^ mDCs, pDCs or a combination of the two, that were stimulated with protamine/RNA and loaded with tumor-associated antigens (Westdorp et al., 2019). The immunotherapy was feasible, well-tolerated with low-grade toxicity and induced functional antigen-specific T cells, which correlated with improved progression-free survival. However, no difference in T cell functionality was observed between the administration of mDCs, pDCs, or mDCs plus pDCs. This strategy is currently under evaluation in a phase I/II clinical trial for stage III melanoma patients (clinicaltrials.gov Identifier: NCT02574377). Additionally, the combined administration of mDCs and pDCs in metastatic endometrial cancer patients is under evaluation (NCT04212377). See Table 1 for an overview of the clinical trials.

**Table 1:** Overview of plasmacytoid dendritic cell vaccination in clinical trials and animal models.

	**Pathology**	**Status**	**General description**	**Intervention and method**	**Outcome measures**	**Results**
NCT01690377 (Tel et al., 2013a)	Melanoma (stage IV)	Phase I Completed (November 2014)	**Participants**: 15 melanoma patients **Type of cells used**: Naturally occurring, autologous pDCs. The maximum feasible dose was 3 × 10^6^ pDCs per injection, which was given to 12/15 patients. **Primary objective**: Generate mature pDCs for a vaccine and determine a safe and effective dosage of pDCs to initiate anti-tumor T cell responses in patients with stage IV melanoma.	**Intervention**: pDCs were activated with inactivated FSME vaccination and loaded with HLA-A2.1-binding tumor antigen-associated peptides (gp100 and tyrosinase) *ex vivo* and administered through intranodal injections. **Vaccination scheme**: Vaccine consists of three intranodal injections given once every 2 weeks. If well-tolerated and no disease progression, then patients were eligible for a maximum of two maintenance cycles of 3 biweekly vaccinations—each with a 6-months interval. DTH^*^: DTH challenge following every vaccine administration.	**Primary endpoint**: Intervention-related toxicity. **Secondary endpoint**: Immunological response.	**Safety**: Vaccine is safe and well tolerated with no signs of severe toxicity. **Immunological effect**: pDCs migrate *in vivo*. Vaccination induces a temporal systemic induction of type I IFNs and activation of T cells responses. PFS/OS^**^: Median OS improved compared with matched controls. PFS was also increased in the vaccine group, however this was not significant.
NCT02692976 (Westdorp et al., 2019)	Prostate cancer (castration resistant, CRPC)	Phase II Completed (March 2019)	**Participants**: 21 chemo-naïve CRPC patients. **Type of cells used**: Naturally occurring, autologous mDCs, pDCs or a combination of mDCs plus pDCs. Vaccinations had a dose of 1–8 × 10^6^ cells for injection. **Primary objective**: To show immunologic efficacy of tumor-peptide loaded natural DC in metastatic castration-resistant prostate cancer patients (mCRPC).	**Intervention**: The DCs were stimulated with protamine/mRNA and loaded with tumor-associated antigens. Patients received maximally nine vaccinations. **Vaccination scheme**: Patients were randomly assigned 1:1:1 to receive one of the three vaccination arms. One cycle of vaccination consisted of 3 biweekly vaccinations administered intranodally in a clinically tumor-free lymph node. 1. Intranodal mDC vaccination (2–5 × 10^6^ cells) 2. Intranodal pDC vaccination (1–3 × 10^6^ cells) 3. Intranodal mDC/pDC vaccination (3–8 × 10^6^ cells) DTH^*^: DTH challenge performed 1–2 weeks after the third vaccination.	**Primary endpoint**: Immunological response by monitoring: 1. Functional response (IFNy^+^) and tetramer (dm^+^) analysis of DTH infiltrating lymphocytes against tumor peptides. 2. Type I IFN gene expression in PBMCs. 3. Proliferative, effector cytokine-, and humoral responses to KLH^****^. **Secondary endpoint**: Safety, feasibility and efficacy of natural DC vaccinations, and the quality of life during treatment.	**Safety**: Vaccinations were well-tolerated with grade 1–2 toxicity (CTCAE^***^). **Immunological effect**: The vaccination enhanced dm^+^ and IFNy^+^ antigen specific T cells. PFS/OS^**^: The overall median radiological PFS was 9.5 months and highest in patients with dm^+^ and IFNy^+^ antigen specific T cells.
NCT04212377	Endometrial cancer	Phase II Recruiting (Estimated completion February 2022)	**Participants**: Estimated enrollment is 8 patients. **Type of cells used**: Naturally occurring, autologous pDCs and mDCs loaded with tumor lysate and MUC1 and surviving PepTivators. **Primary objective**: To investigate the hypothesis that pDCs and mDCs are optimal for vaccines to combat cancer. To investigate the hypothesis that the combination of mDC and pDC may induce stronger anti-tumor immune responses as compared to pDC or mDC alone, or moDC.	**Intervention**: Metastatic endometrial cancer (mEC) patients who receive Carboplatin/Paclitaxel chemotherapy will get the vaccine. **Vaccination scheme**: The chemotherapy is given in a weekly schedule (weeks 1–3 and 5–7), and the patients will receive the vaccine by intranodal injection in week 8. DTH^*^:	**Primary endpoint**: The anti-tumor response in the mEC patients evaluated by type I IFN expression, response to KLH (proliferative, effector cytokine, and humoral responses) **Secondary endpoint**: Safety by number of participants with adverse effects and change from baseline in pain scores.	**Safety**: **Immunological effect**: PFS/OS^**^:
NCT02574377	Melanoma (stage III)	Phase I/II Unknown recruitment status (Estimated completion December 2019)	**Participants**: Estimated enrollment is 30 patients. **Type of cells used**: Naturally occurring, autologous mDCs and pDCs, or combined, loaded with. tumor peptides. **Primary objective**: To the immunogenicity of combined adjuvant mDC and pDC vaccination vs. adjuvant mDC or pDC vaccination alone in stage III melanoma patients.	**Intervention**: 2 biweekly vaccinations of intranodal injections with pDC, mDC or combination of pDC and mDC. **Vaccination scheme**: Three-arm study, where stage lll melanoma patients will receive: 1. A: pDC (*n* = 10), 2. B: mDC (*n* = 10), 3 C: Combined pDC/mDC (*n* = 10). If patients remain disease free, the cycle will be repeated up to three times with a 6 months interval. DTH^*^: A challenge with peptide loaded blood DC is performed from which biopsies are taken for T cell analysis.	**Primary endpoint**: Immune response evaluated by type I IFN expression, response to KLH (proliferative, effector cytokine and humoral responses) and functional response and tetramer analysis of DTH infiltrating T cells against tumor peptides.**Secondary endpoint**: Distribution of mDC and pDC in the lymph node, toxicity, quality of life, PFS, and OS.	**Safety**: **Immunological effect**: PFS/OS^**^:
NCT03970746	Non-Small Cell Lung Cancer (NSCLC)	Phase I/II Recruiting (Estimated completion August 2022)	**Participants**: Estimated enrollment is 66 patients. **Type of cells used**: Vaccine is based on the pDC cell line HLA-A^*^02:01 that is lethally irradiated and then pulsed with peptides from target tumor antigens. **Primary objective**: To assess the tolerability, the immunogenicity and the preliminary clinical activity of the therapeutic cancer vaccine, PDC^*^lung01, either alone or associated with anti-PD-1 treatment in patients with NSCLC.	**Intervention**: Administration of the vaccine either alone (cohort A) or together with Pembrolizumab (anti-PD-1, cohort B). **Vaccination scheme**: Four-arm vaccination study, consisting of: 1. A1: Low dose PDC^*^lung01 as single agent or during maintenance treatment by Pemetrexed (chemotherapy). 2. A2: High dose PDC^*^lung01 as single agent or during maintenance treatment by Pemetrexed (chemotherapy). 3. B1: Low dose PDC^*^lung01 added to Pembrolizumab. 4. B2: High dose PDC^*^lung01 added to Pembrolizumab. Cohort A1/2 will receive vaccine at six visits; subcutaneously followed by intravenous route. Cohort B1/2 will receive first vaccine injection 48 h after first infusion of anti-PD-1. The fourth vaccine injection will occur within 48 h after the second infusion of Pembrolizumab. DTH^*^:	**Primary endpoint**: Occurrence of dose-limiting toxicity related to administration of the vaccine. **Secondary endpoint**: Evaluation of adverse events induced by the vaccination. Evaluation of the cellular and humoral immunological response to the vaccine.	**Safety**: **Immunological effect**: PFS/OS^**^:
Martinet et al. (2012)	Hepatitis B Virus (HBV)	NOD/SCID β2m^−/−^ mice	**Type of cells used**: Vaccine is based on the human pDC cell line HLA-A^*^02:01 that is lethally irradiated and then pulsed with HBV peptides or control peptides.	**Design:** mice are reconstituted with 50 × 10^6^ PBMCs from a resolved HBV patient and xenotransplanted with 25 × 10^6^ human HBV-transfected or untransfected hepatocytes. **Vaccination scheme:** Mice were treated with pDCs 3 days before or 3 days after challenge with hepatocytes. Mice received 5 × 10^6^ pDCs per vaccination injected at the peritoneal lavage. One injection per week, two injections in total.	**T cells:** After two vaccination, HBV-specific T cells were analyzed via tetramer labeling at the site of injection, draining lymph node, spleen and blood. **Hepatocytes**: size was measured every 2–3 days **Viral load**: measured in serum	Vaccination before and after hepatocyte challenge amplified HBV-specific T cells, inhibited expansion of transfected hepatocytes, and reduced systemic viral load.
Remer et al. (2007)	*Leishmania major* (*L. major*)	BALB/c mice	**Type of cells used**: Splenic murine pDCs pulsed with *L. major* lysates or no lysate	**Design:** 1 week or 4 weeks after pDC vaccination, mice are challenged with *L. major* intradermal in the footpad. Five weeks after challenge, splenic T cells from protected mice are transferred to naïve mice that were then challenged. **Vaccination scheme:** single vaccination in the tail vein of 5 × 10^4^ pDCs.	**Footpad:** swelling, inflammation, ulceration **Parasites:** Parasitic burden in footpad 6 weeks after challenge **Cytokine profile**: LN and spleen cells 6 weeks after challenge (IFNγ, IL-4, IL-10) **IgG subclass profile:** *L. major* specific IgG1 and IgG2a antibodies 5 weeks after challenge.	A single vaccination and adoptive T cell transfer of vaccinated mice onto naïve mice protected against *L. major* infection. Protection was not accompanied by a Th1 cytokine profile but protected animals had lower ratios of IgG1 to IgG2a in sera.

* *Delayed-type hypersensitivity challenge*.

** *Progression free survival/Overall survival*.

*** *Common terminology criteria for adverse events*.

**** *KLH, Keyhole Limpet Hemocyanin (a protein providing T cell help)*.

A limiting factor in using peripheral blood-derived pDCs is the number of pDCs available for vaccination. Only a small fraction of peripheral blood mononuclear cells (PBMCs) consists of pDCs (<1%). In the two before-mentioned clinical trials, leukapheresis was used to obtain pDCs and a maximum of three million pDCs could be administered per infusion, which was repeated on three occasions (Tel et al., 2013a; Westdorp et al., 2019). Overcoming the limitation in pDC numbers would greatly improve the application of pDCs as cell-based therapy. One way to get around this is to use an allogeneic pDC cell line. Allogeneic HLA-A^*^02:01 pDCs can induce melanoma antigen-specific and functional cytotoxic T cell responses *ex vivo* and have been shown to inhibit tumor growth in a humanized mouse model (Aspord et al., 2010, 2012). The safety and tolerability of using the irradiated HLA-A^*^02:01 pDC cell line loaded with four melanoma peptides (GeniusVac-Mel4) is currently under evaluation in a phase I clinical trial (NCT01863108). Similarly, a pDC cell line (PDC^*^lung01, PDC^*^line Pharma) is currently in a phase I/II study for the treatment of non-small-cell lung cancer (NCT03970746). However, the allogeneic pDC vaccine approach has some challenges; it is restricted to HLA-A2 patients and irradiation of the cells impairs the possibility to initiate an innate immune response via the secretion of IFN.

One possibility to obtain a continuous source of pDCs applicable for vaccination is to generate them *ex vivo* from hematopoietic stem cells. Cord blood CD34^+^ hematopoietic stem and progenitor cells (HSPC) have been shown to be suitable for the differentiation into functional pDCs *in vitro* (Blom et al., 2000; Chen et al., 2004; Olivier et al., 2006; Demoulin et al., 2012; Thordardottir et al., 2014) and can yield clinically relevant cell numbers: up to 81 (±20) pDCs per single HSPC (Laustsen et al., 2018). CD34^+^ stem cells can also be isolated from peripheral blood after mobilization with G-CSF and the generated pDCs can induce Ag-specific activation of autologous CD8^+^ memory T cells *ex vivo* (Thordardottir et al., 2017). Although using autologous stem cell-derived pDCs for vaccination is a promising avenue for personalized pDC therapeutics, the HSPC differentiation into pDCs still requires long-term culturing, implying that the field still needs to make several advancements before it has clinical potential.

### pDCs as Therapeutic Vaccine for the Treatment of Infectious Diseases

As of today, there are two reports that describe the use of pDCs as therapeutic vaccine for the treatment of an infectious disease. In the first study, the HLA-A^*^02:01 pDC line was used for the treatment of Hepatitis B Virus (HBV) (Martinet et al., 2012). Immunodeficient NOD/SCID β2m^−/−^ mice, reconstituted with HBV patient's PBMCs and xenotransplanted with human HBV-transfected hepatocytes, received two vaccinations of irradiated HBV-peptide pulsed pDCs per treatment. Vaccination elicited HBV-specific T cells that were able to lyse the transfected hepatocytes and reduce systemic viral load. In the second study, pDCs were used to vaccinate BALB/c mice to provide protection against the parasitic infection *Leishmania major* (*L. major*) (Remer et al., 2007). Mice received a single dose of splenic murine pDCs that were pulsed with *L. major* lysate. Vaccination provided complete protection when mice were challenged 1 or 4 weeks after vaccination. Additionally, adoptive T cell transfer of protected mice onto naïve susceptible mice provided complete protection to *L. major* challenge.

These studies show that pDC vaccination can provide protection against two different infectious diseases in murine models.

### HIV Infection Negatively Impacts pDC Numbers and Functionality

Similar to the decline in CD4^+^ T cell counts following acute HIV infection, a decline in circulating pDCs can be observed. Upon initiation of antiretroviral therapy (ART) the pDC numbers can increase but are not fully restored (Donaghy et al., 2001; Chehimi et al., 2002; Barron et al., 2003; Finke et al., 2004; Almeida et al., 2005; Kamga et al., 2005; Lichtner et al., 2008; Centlivre et al., 2011; Boichuk et al., 2015; Marquez-Coello et al., 2019). Additionally, pDCs that remain in circulation seem to be dysfunctional (further discussed below).

The reason for the decline in pDC numbers is not fully understood. PDCs express CD4, CCR5 and CXCR4, which theoretically makes them susceptible to infection with HIV. When isolated from peripheral blood or the thymus, pDCs can indeed be infected with both X4 and R5-tropic HIV *in vitro* (Patterson et al., 2001; Fong et al., 2002; Yonezawa et al., 2003; Lore et al., 2005; Smed-Sorensen et al., 2005; Evans et al., 2011). Whether this occurs *in vivo* is not clear, as conflicting observations have been reported. HIV DNA has been detected in pDCs isolated from people living with HIV (PLWH) and pDCs from the tonsil have been found to be positive for the CA-p24 protein (Fong et al., 2002; Donaghy et al., 2003; Centlivre et al., 2011). In contrast, others have not been able to demonstrate the presence of HIV RNA or DNA in peripheral blood pDCs, although this has been explored in samples from PLWH on suppressive ART (Otero et al., 2003) whereas in the other studies the PLWH were therapy naïve.

Yet, the infection of pDCs by HIV seems counterintuitive since they are the major producers of type I IFNs, and IFNs are well-known to have a potent anti-viral effect by increasing the expression of virus restriction and inhibition factors (Kluge et al., 2015; Colomer-Lluch et al., 2018). However, the HIV-induced IFN production by pDCs is delayed and is up to 20-fold lower than IFN production induced by Influenza, Sendai, and HIV-2 (Lo et al., 2012). Furthermore, pDCs do express the restriction factors SAMHD1 (Bloch et al., 2014), tetherin/CD317 (Tavano et al., 2013), and APOBEC3G (Wang et al., 2008) but, similar to other cell types, the expression of these is enhanced by IFN signaling. Thus, it remains a possibility that pDCs can be infected with HIV prior to this delay in IFN production and subsequent delay in viral restriction factor expression. PDC depletion may also occur in people living with HIV-2 in the absence of detectable viremia (Cavaleiro et al., 2009). This indicates that mechanisms other than direct viral infection determine the pDC depletion during persistent infections. A second potential mechanism is that type I IFN negatively controls pDC cell numbers as this has been observed using mice. Viral infection induced the upregulation of pro-apoptotic molecules in pDCs in a type I IFN-dependent manner, resulting in caspase activation and subsequent pDC death (Swiecki et al., 2011). A third possibility to explain the decrease in pDC cell numbers in the periphery is that pDCs no longer circulate. HIV-activated pDCs enhance expression of the lymphoid homing receptor CCR7 and during progressive infection or in the absence of ART, they have been reported to accumulate in LNs and lymphoid tonsillar tissue (Fonteneau et al., 2004; Schmidt et al., 2005; Herbeuval et al., 2006; Lehmann et al., 2010). Another study reported that in the presence of ART, pDCs also home to the LNs but compared to people living without HIV, the pDC numbers in LNs were significantly reduced (Biancotto et al., 2007). This indicates that both circulatory and LN pDC numbers decline, but that the decrease of pDCs in circulation is not necessarily due to homing to lymphoid tissue.

Besides reduced cell numbers, pDCs are also dysfunctional in PLWH and persistently produce low levels of IFNs (O'Brien et al., 2011). During ART, the TLR7/8 response appears to remain intact (Chang et al., 2012; Bam et al., 2017; Tsai et al., 2017) but IFN production upon TLR9 signaling is reduced, potentially due to continuous HIV-induced activation of pDCs *in vivo* or the interaction of CD40 with CD40L (Feldman et al., 2001; Tilton et al., 2008; Cavaleiro et al., 2009; Donhauser et al., 2012). Additionally, pDCs in the blood of PLWH display an exhausted phenotype and this may interfere with TLR signaling and subsequent IFN production (Cavaleiro et al., 2009; Schwartz et al., 2017; Font-Haro et al., 2018). However, administration of the TLR9 agonist MGN1703 to PLWH on ART enhances pDC activation, as measured by activation markers CD86 and CD40. Plasma IFNα-2a levels increased as well, indicating that some TLR9 responsiveness remains or that other cells may produce IFN in response to MGN1703 (Vibholm et al., 2017).

### Would There Be Benefit in Restoring pDC Cell Numbers With a Functional IFN Response?

There are observations that indicate a beneficial role of maintaining pDC numbers during HIV infection. First, PLWH on ART with a low baseline pDC blood count are more likely to have an increase of HIV-RNA compared to individuals with high pDC counts during a 30 months follow-up (Lichtner et al., 2008). Second, elite controllers and long-term non-progressors (LTNPs) have preserved pDC counts and functionality (Almeida et al., 2005; Machmach et al., 2012). It is unclear whether the pDCs from elite controllers are better equipped to suppress viral replication or if the pDC cell numbers are maintained because the viral load is suppressed by other immune cells, like CTLs or natural killer (NK) cells. Third, expansion of pDCs during acute infection delays onset of viremia and reduces HIV replication in humanized mice (Pham et al., 2019).

### pDCs Can Potentially Control HIV via Three Mechanisms

**Figure 1:**
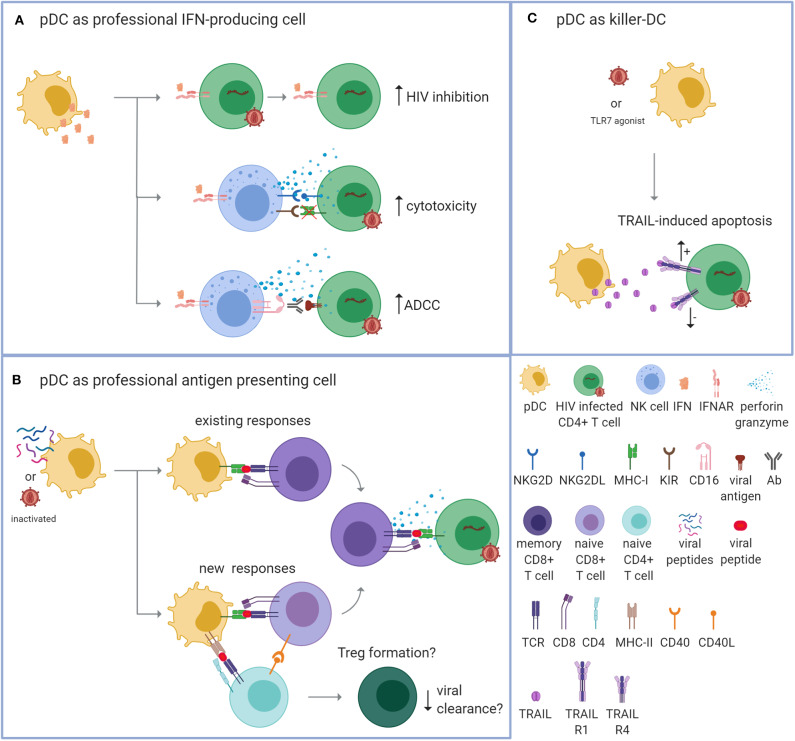
Plasmacytoid dendritic cells can potentially control HIV via three mechanisms. Plasmacytoid dendritic cells (pDCs) can contribute to the suppression of virus replication by fulfilling three different functions. **(A)** As a professional interferon (IFN)-producing cell. PDCs can produce vast amounts of IFNs upon TLR7/9 signaling. IFNs can directly inhibit HIV replication by enhancing the expression of virus restriction and inhibition factors within the CD4^+^ T cell (top; increased HIV inhibition). Additionally, IFNs can provide an immunostimulatory environment that enhances the cytotoxic function of natural killer (NK) cells. Apoptosis of HIV-infected cells can be induced by the release of cytotoxic perforins and granzymes after detecting reduced expression of MHC-I molecules in combination with activating signals, such as the binding of NKG2D to NKG2DL (middle; enhanced cytotoxicity). Apoptosis can also be induced by detecting the Fc tail of an antibody (Ab) that is bound to a viral antigen on the surface of the infected cell [bottom; enhanced antibody-dependent cellular cytotoxicity (ADCC)]. **(B)** As a professional antigen presenting cell (APC). PDCs can process endogenous and exogenous antigens for the antigen-specific stimulation of T cells. PDCs could be loaded with autologous inactivated HIV or HIV peptides that are presented on MHC-I molecules to activate existing HIV-specific memory CD8^+^ T cells (top; existing responses). Upon repeated exposure, this strategy could be applied to induce new responses by activating naïve CD8^+^ T cells with the help of CD4^+^ T cells (bottom; new responses). The activated HIV-specific CD8^+^ T cells are then able to recognize and eliminate HIV-infected cells via a cognate TCR-MHC interaction. PDCs may also induce the formation of regulatory T cells (Tregs) that could potentially suppress immune activation and counteract viral clearance. **(C)** As a “killer-pDC.” TLR7-stimulated or HIV-exposed pDCs can obtain cytotoxic properties through the expression and secretion of TNF-related apoptosis-inducing ligand (TRAIL) that can bind to the apoptosis-transmitting TRAIL receptor 1 (R1). However, HIV-infected CD4^+^ T cells from PLWH who receive antiretroviral treatment express the TRIAL R1 receptor and the decoy receptor TRAIL R4, which makes the T cells likely resistant to pDC-mediated killing.

PDCs can contribute to suppression of virus replication by fulfilling three different functions: (i) as a professional IFN-producing cell; (ii) as a professional APC and; (iii) as a “killer-pDC” with cytotoxic properties (Figure 1). Each mechanism is discussed individually below.

#### Controlling HIV as a Professional IFN-Producing Cell

PDCs produce abundant type I IFNs (Kadowaki et al., 2000) and type III IFNs (Yin et al., 2012) when sensing viral products such as single stranded RNA by TLR7 or unmethylated DNA molecules by TLR9, reviewed in Swiecki and Colonna (2015). In the context of HIV, pDCs produce type I IFNs in response to cell-free HIV and to HIV infected cells; both require endocytosis and likely initiate the TLR7 signaling pathway (Beignon et al., 2005; Schmidt et al., 2005). IFNs function as the first line of defense against viruses because they have a broad antiviral effect. IFNs can induce inflammation, activate specific immune cells including NK cells, CD8^+^ T cells, and macrophages, and prime antigen-specific responses, reviewed in Hoffmann et al. (2015) and Kotenko and Durbin (2017). IFNs can also directly inhibit HIV replication by enhancing the expression of virus restriction and inhibition factors, reviewed in Colomer-Lluch et al. (2018) (Figure 1A).

However, chronic inflammation and long-term exposure to IFNs have clear detrimental effects (Acchioni et al., 2015). In the context of HIV, the timing of IFN seems to be critical, as demonstrated in a study with SIV-infected rhesus macaques. Blocking type I IFN signaling during acute SIV infection enhanced virus replication and CD4^+^ T cell loss, whereas administering IFNα-2a during acute SIV infection induced an antiviral state and limited viral spread. However, when IFNα-2a treatment continued, cells became unresponsive to IFN, resulting in decreased viral inhibition and subsequent enhanced CD4^+^ T cell loss (Sandler et al., 2014). Thus, ideally pDCs should produce IFNs for a limited time only.

In addition to upregulating virus restriction and inhibition factors, IFNs provide an immunostimulatory environment for other immune cells (Gonzalez-Navajas et al., 2012). This is particularly apparent for the cytolytic capacity of natural killer (NK) cells (Figure 1A). IFNs produced by stem-cell derived pDCs can trigger NK cell activation and increase the capacity of NK cell-mediated killing of acute lymphoblastic leukemia (Diaz-Rodriguez et al., 2017). Similarly, IFN produced by TLR9-stimulated peripheral blood pDCs can enhance NK-mediated lysis of autologous CD4^+^ T cells infected with HIV *in vitro* (Tomescu et al., 2007). Additionally, NK-mediated antibody-dependent cellular cytotoxicity (ADCC) via autologous and heterologous HIV serum antibodies can be enhanced by IFNα (Lee et al., 2015; Tomescu et al., 2017). ADCC is a mechanism of cell-mediated cytotoxicity where the effector cell, here the NK cell, lyses a target cell via the recognition of an antibody that is bound to a viral antigen on the target cell. Via CD16, the effector cell binds the Fc portion of the antibody followed by the release of cytotoxic factors (Perussia and Trinchieri, 1984; Perussia et al., 1984).

#### Controlling HIV as an Antigen-Presenting Cell

Multiple studies have shown that pDCs can process and present endogenous and exogenous antigens on MHC I and II molecules and induce antigen-specific activation of both CD8^+^ and CD4^+^ T cells (Fonteneau et al., 2003; Benitez-Ribas et al., 2006; Young et al., 2008; Tel et al., 2010, 2013b). Important for therapeutic purposes, pDCs can be loaded with synthetic long and short peptides to trigger CD4^+^ and CD8^+^ T cell responses *in vitro* (Aspord et al., 2014) and *in vivo* (Tel et al., 2013a; Westdorp et al., 2019). This suggests that, similar to monocyte-derived DCs (moDCs), pDCs could be loaded with HIV antigens *in vitro* to enhance T cell responses *in vivo* (Figure 1B). To date, 17 clinical trials using moDC vaccination for HIV infection have been published and the clinical outcomes were variable, reviewed in da Silva et al. (2018). The studies all had in common the use of moDCs, but differed regarding moDC preparation and maturation, HIV antigen, route of administration, the number of cells administered per dose, and the number of doses administered. Regardless of these variations, all trials were safe and well tolerated. Seven of the seventeen studies demonstrated a prolonged suppression of HIV RNA in plasma and this correlated with HIV-specific T cell responses. In these studies the moDCs were exposed to autologous aldrithiol-2-inactivated HIV (Lu et al., 2004), autologous heat-inactivated HIV (Garcia et al., 2005, 2011, 2013), autologous inactivated HIV-infected apoptotic cells (Macatangay et al., 2016), or HIV peptides (Kloverpris et al., 2009; Levy et al., 2014). If these strategies are appropriate for pDC vaccination remains to be determined.

Besides inducing inflammatory T cells, pDCs have been reported to induce the formation of regulatory T cells (Tregs) and expression of indoleamine 2,3-dioxygenase (IDO) by pDCs is one of the reported mechanisms (Chen et al., 2008). Additionally, HIV-stimulated pDCs have been reported to differentiate naïve CD4^+^ T cells into Tregs (Manches et al., 2008). Tregs suppress immune activation and limit viral clearance, reviewed in Kleinman et al. (2018). Thus, in the context of using pDCs as a therapy to induce elimination of HIV-infected cells, the induction of Tregs would likely be undesirable. However, whether pDCs exposed to inactivated HIV or HIV peptides also induce Treg formation remains to be determined.

#### Controlling HIV as a Killer-pDC

In a mouse model for melanoma, TRL7-stimulated pDCs have been shown to directly kill tumor cells via the secretion of TNF-related apoptosis-inducing ligand (TRAIL) and granzymes (Drobits et al., 2012). Similarly, HIV-exposed pDCs also gain cytotoxic properties, thereby creating so-called “killer-pDCs,” which can induce apoptosis of CD4^+^ T cell lines via the TRAIL pathway (Hardy et al., 2007; Chehimi et al., 2010; Barblu et al., 2012; Figure 1C). This seems to have great potential for eliminating virus-infected cells but expression of the apoptosis-transmitting TRAIL receptor 1 (R1) is not restricted to HIV-infected cells and seems to be the result of chronic immune activation, possibly resulting in the killing of bystander T cells as well (Stary et al., 2009). CD4^+^ T cells express TRAIL R1 during detectable viremia but initiation of ART enhances expression of the decoy receptor TRAIL R4, making the T cells resistant to pDC-mediated killing (Stary et al., 2009; Chehimi et al., 2010). Unless expression of the decoy TRAIL R4 can be downregulated on HIV-infected cells, the TRAIL-expressing pDC approach seems like a rather unattractive strategy to treat HIV infection because it would require pausing therapy and would not be limited to HIV-infected cells only.

## Conclusions and Future Directions

In summary, pDCs are multifaceted cells and could have the potential to enhance immune function to inhibit HIV replication and clear infected cells (Figure 1). Similar to moDCs, pDCs have the capacity to function as APC and activate existing memory T cells or induce the activation of naïve T cells. Whether pDCs can achieve this *in vivo* after exposure to HIV Ag needs to be evaluated. Additionally, a key point to address in this regard is the pDCs' capacity to induce Tregs. If pDCs were to be used as therapeutic vaccine, it would be critical to evaluate the formation of Tregs as these anti-inflammatory cells could limit viral clearance.

IFNs are key mediators of an anti-viral response and although pDCs are potent producers of type I IFNα it is important to emphasize that IFNα comprises 13 different subtypes. They all bind to the same receptor but have distinct biological activities, and display differences in their HIV inhibiting properties (Gibbert et al., 2013; Harper et al., 2015; Lavender et al., 2016). Depending on the type of TLR stimulation, pDCs can produce a broad range of IFNα subtypes (Puig et al., 2012; Harper et al., 2015). There may be a benefit in using pDCs as a tool to deliver a broad range of IFNs, rather than administering a single subtype, which is the current approach with pegylated (peg)IFNα-2a. An additional benefit of using pDCs for the production of IFNs could be the local delivery of IFNs. As pDC can travel to lymphoid organs and other peripheral tissues, this may allow for more local and targeted delivery of IFNs rather than systemic administration via intravenous injection.

Lastly, there are currently three possibilities to obtain pDCs for therapeutic vaccination. pDCs can be obtained from peripheral blood via leukapheresis, generated from stem cells obtained from peripheral blood after mobilization, or provided as irradiated pDC cell lines. Each source has its advantages and disadvantages, and the choice may depend on the function that the pDCs are required to fulfill in order for the therapy to be effective. As APCs, pDCs would ideally be from an autologous source, but could also be from an allogeneic HLA-matched source.

However, as professional IFN-producing cells there would be no requirement for HLA-matching and it would perhaps be possible to generate a pDC vaccine from allogeneic origin. While this is an intriguing avenue of research, there are several advancements required within the field before pDC vaccination is able to reach its clinical potential for the treatment of HIV.

## Author Contributions

RS and MJ conceptualized the idea for this manuscript. RS outlined the manuscript and wrote the first draft. JE conceptualized Table 1 and contributed together with MJ to editing the manuscript. All authors approved the final version.

## Conflict of Interest

The authors declare that the research was conducted in the absence of any commercial or financial relationships that could be construed as a potential conflict of interest.
